# Community voices: NIH working toward inclusive excellence by promoting and supporting women in science

**DOI:** 10.1038/s41467-022-28665-2

**Published:** 2022-03-25

**Authors:** Kelly G. Ten Hagen, Carrie Wolinetz, Janine A. Clayton, Marie A. Bernard

**Affiliations:** 1 National Institute of Dental and Craniofacial Research, National Institutes of Health, Bethesda, MD USA; 2 Office of Science, Technology, and Policy, White House, Washington, DC USA; 3 Office of Research on Women’s Health, Office of the Director, National Institutes of Health, Bethesda, MD USA; 4 Office of the Chief Officer for Scientific Workforce Diversity, Office of the Director, National Institutes of Health, Bethesda, MD USA

**Keywords:** Society, Careers, Institutions

## Abstract

The U. S. National Institutes of Health is committed to addressing gender discrimination and fostering inclusive excellence, which is critical for the advancement of creativity and innovation in science. Strategies and processes aimed at achieving these goals are discussed.

## Working toward inclusive excellence by promoting and supporting women in science

While it is not news that women are underrepresented in many fields of science and that gender bias exists in the research enterprise, the data continue to shock. The American Association of Medical Colleges (AAMC) reports that while women in the United States (U.S.) received nearly half of medical school degrees, women accounted for only 18 percent of key leadership positions, such as department chairs or deans^1^. At a recent National Academies of Sciences, Engineering, and Medicine (NASEM) workshop, it was noted that U.S. women were subject to a gender gap in salaries and that women of color, in particular, are dramatically underrepresented across all career stages (13 percent of medical faculty) with little improvement over nearly a decade^2^. Even women who have achieved success in medical research have had to do so while coping with a hostile work environment in which sexual harassment and gender discrimination are common^3^. Unfortunately, the scientific enterprise is just a reflection of a broader society that diminishes the status and achievements of women, as recently demonstrated by an op-ed in which it was suggested that Dr. Jill Biden, First Lady of the USA, discard her professional title. The outrage^4^ sparked by that piece gave voice to professional women weary of being addressed by their first names, as their male colleagues are accorded their due respect and called “Doctor”.

There is reason to redouble our efforts to support and promote women in science. The COVID-19 global pandemic has had a disproportionately negative effect on women, who bear a greater burden of caregiving and household responsibilities than their male colleagues^5^. Troubling early evidence of the pandemic’s impact suggests losses in productivity for women scientists—in particular, a disproportionate impact on women with caregiving responsibilities^6^—raising concerns of setbacks in any progress made toward gender equity^7^. Moreover, despite a sense that gender equity in science has taken on a new urgency in recent years, constant vigilance is required to eliminate bias in the workplace. It is equally important to develop solid methods that integrate and address the existence of implicit bias when carrying out and evaluating meta-research on scientific activities or there is a risk to reach potentially harmful conclusions. You need to look no farther than the recently retracted publication that erroneously suggested that being mentored by a woman harmed the mentee’s career^8,9^. It is still far too common for views suggesting the inferiority of women scientists to find purchase in our system. Likewise, it is still far too common to find clear inequities in salaries, resources, space, and opportunities for advancement. The national reckoning on racial equity and justice has increased the visibility of women of color in science, who encounter an even greater set of challenges and obstacles at the confluence of racial and gender discrimination^10^.

**Fig. 1:**
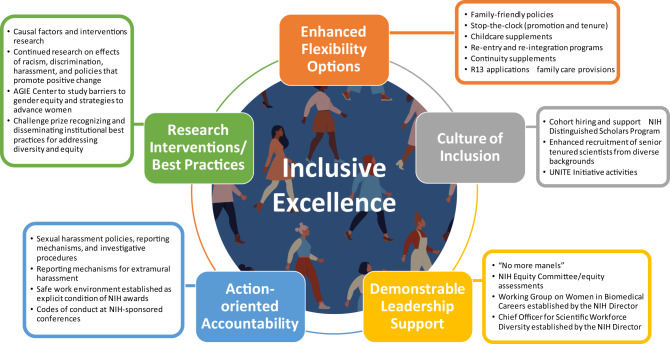
A multifaceted/coordinated approach to advance inclusive excellence. There are multiple complementary components of inclusive excellence that interact to foster a diverse scientific ecosystem. Support of each component and coordinated action is essential to full inclusion of women in the scientific workforce.

The National Institutes of Health (NIH) has made strides in redressing gender discrimination and fostering inclusive excellence in its intramural and extramural enterprises (Fig. 1). But there is still much work to do.

For example, the agency has taken a strong stand against sexual harassment, including gender harassment and bullying, through action-oriented accountability. Intramurally, there now exist transparent policies and investigative procedures, as well as anonymous and centralized reporting systems, to address this substantial cultural barrier for women in science (https://www.nih.gov/anti-sexual-harassment) As part of our ongoing efforts to combat sexual harassment and other inappropriate behaviors in the extramural world, we have taken steps to ensure safe work environments, including expectations that institutions notify NIH when there are concerns about safety serious enough to change the status of a PI on a grant (NOT-OD-20-124)^11^. We have made a safe work environment, free of harassment, an explicit condition of all awards, and have provided mechanisms where harassment can be reported to the Office of Extramural Research^12^. NIH has also developed guidelines for creating safe and inclusive environments at NIH-funded conferences (https://grants.nih.gov/grants/guide/notice-files/NOT-OD-21-053.html).

NIH leadership has publicly and visibly supported women in science, an example being former NIH Director Francis Collins’s declaration that he would no longer participate in all male panels, or “manels” (https://www.nih.gov/about-nih/who-we-are/nih-director/statements/time-end-manel-tradition). Additionally, NIH leadership established the NIH Equity Committee (https://diversity.nih.gov/programs-partnerships/nih-equity-committee) to review hiring, resource allocation, and promotion procedures and policies within the NIH Intramural Research Program (IRP). The Women Scientists Advisors Committee, which spearheaded the anti-harassment efforts within the NIH, was also established by NIH leadership. The Chief Officer for Scientific Workforce Diversity (which supports diversity and inclusion) as well as the Working Group on Women in Biomedical Careers (which aims to explore new approaches to recruit and retain women in science) were both established by the NIH Director to support women in biomedicine.

The NIH IRP has also modeled practices for hiring cohorts who are committed to NIH’s values of diversity, equity, and inclusion, through the NIH Distinguished Scholars Program (https://diversity.nih.gov/programs-partnerships/dsp) and the Stadtman faculty recruitment program (https://irp.nih.gov/careers/trans-nih-scientific-recruitments/stadtman-tenure-track-investigators). But with these advances, we need to acknowledge there is still a long way to go, particularly with regard to the underrepresentation of women and other historically marginalized groups in both science and leadership within the NIH. A new effort at the NIH, known as UNITE, has been established to address racial inequities that have prevented certain groups from participating in the scientific enterprise, including women from racial and ethnic groups underrepresented in U.S. biomedical science^13,14^. The initiative includes a commitment to recruit more senior tenured scientists from underrepresented groups into the NIH IRP. There is additionally a commitment to identify and remove barriers in policies and practices that contribute to the underrepresentation of groups due to bias.

The NIH is bringing new and creative approaches to overcoming the inequities experienced by women in biomedical research. It has expanded the NIH re-entry program to provide a re-integration pathway for scientists including pre-doctoral students whose careers have been derailed by harassment or discrimination (https://grants.nih.gov/grants/guide/notice-files/NOT-OD-21-134.html). To ensure evidence-based approaches, the NIH is funding research to understand the impacts of racism, discrimination, and harassment on the biomedical workforce and identify ways to intervene against them (https://grants.nih.gov/grants/guide/pa-files/PAR-19-295.html). And combatting harassment has been identified as an area of interest in a recently issued FOA that invites, for the first time, applications on interventions to address harassment (https://grants.nih.gov/grants/guide/notice-files/NOT-OD-21-068.html). The Office of Research on Women’s Health (ORWH) is working with the National Institute for Diabetes and Digestive and Kidney Diseases (NIDDK) to establish a coordination center for Advancing Gender Inclusive Excellence (AGIE) “aimed at investigating barriers, strategies, approaches, and interventions to help women attain leadership positions in many areas of science” (https://grants.nih.gov/grants/guide/notice-files/NOT-OD-21-051.html). The NIH Working Group on Women in Biomedical Careers (WG) created a new competition named the Enhancing Faculty Gender Diversity in Biomedical and Behavioral Science Prize (https://orwh.od.nih.gov/about/director/messages/nih-launches-challenge-prize-help-address-gender-diversity-and-equity) to recognize institutions that have successfully and systemically addressed gender diversity and equity issues among faculty members in biomedical and behavioral sciences. Its aim is not only to increase gender diversity in these fields, but also to promote the broad dissemination and adoption of replicable, evidence-based institutional approaches to promoting gender diversity. To focus on critical transition periods in career trajectories, ORWH also partnered with the WG and OER to create supplements to promote continuity and retention of K awardees and first-time research project grant recipients to apply for flexible funding to maintain research productivity when they experience a critical life event such as childbirth, adoption, and primary caregiving responsibility for an ill immediate family member (https://grants.nih.gov/grants/guide/notice-files/NOT-OD-20-054.html). Additionally, in March 2021, the NIH announced the provision of childcare support for recipients of Ruth L. Kirschstein National Research Service Awards (NRSA) (https://grants.nih.gov/grants/guide/notice-files/NOT-OD-21-074.html) and extensions of the timeline for applying for K awards, in an effort to enhance flexibility during a time when many are struggling with additional burdens brought on by the pandemic.

While these collective strategies are important and represent positive progress, continued effort is needed to achieve a culture of true equity and inclusion, where women scientists are not marginalized, diminished, or harassed, in any professional setting or in the public eye. This redoubling of efforts is particularly important given the disproportionate impact of the pandemic on women, which is likely to continue well into the future. And this will require continuous assessment of environments, policies, and practices, as well as flexible multipronged approaches to address inequities and obstacles as they are identified. NIH is committed to achieving gender equity across the research enterprise and disrupting the environments that serve as barriers to women and other marginalized groups. This is not just the right thing to do, it is critical to our mission to fund and conduct the best science to improve human health. There is abundant evidence^15^ that a diverse scientific workforce results in better science and increased innovation within the biomedical enterprise. As representatives of the largest USA funder of biomedical research, we owe it to the American taxpayers to ensure full participation of women and underrepresented groups in research. The same principle applies for all involved in this research across the globe. The health of our world community depends on it.
